# Applying the socio-ecological model to understand factors associated with sugar-sweetened beverage behaviours among rural Appalachian adolescents

**DOI:** 10.1017/S1368980021000069

**Published:** 2021-08

**Authors:** Brittany A McCormick, Kathleen J Porter, Wen You, Maryam Yuhas, Annie L Reid, Esther J Thatcher, Jamie M Zoellner

**Affiliations:** 1 Department of Public Health Sciences, UVA Cancer Center Research and Outreach Office, University of Virginia, 16 East Main Street, Christiansburg, VA 24073, USA; 2 Department of Public Health Sciences, School of Medicine, University of Virginia, Charlottesville, VA 22903, USA; 3 Department of Nutrition and Food Studies, Syracuse University, Syracuse, NY 13244, USA; 4 Department of Population Health, University Hospitals, Cleveland, OH 44106, USA

**Keywords:** Sugar-sweetened beverages, Socio-ecological model, Rural Appalachia, Adolescents

## Abstract

**Objective:**

The objective of the current study was to identify factors across the socio-ecological model (SEM) associated with adolescents’ sugar-sweetened beverage (SSB) intake.

**Design:**

This cross-sectional study surveyed adolescents using previously validated instruments. Analyses included descriptive statistics, ANOVA tests and stepwise nonlinear regression models (i.e., two-part models) adjusted to be cluster robust. Guided by SEM, a four-step model was used to identify factors associated with adolescent SSB intake – step 1: demographics (i.e., age, gender), step 2: intrapersonal (i.e., theory of planned behaviour (attitudes, subjective norms, perceived behavioural control, behavioural intentions), health literacy, media literacy, public health literacy), step 3: interpersonal (i.e., caregiver’s SSB behaviours, caregiver’s SSB rules) and step 4: environmental (i.e., home SSB availability) level variables.

**Setting:**

Eight middle schools across four rural southwest Virginia counties in Appalachia.

**Participants:**

Seven hundred ninety seventh grade students (55·4 % female, 44·6 % males, mean age 12 (sd 0·5) years).

**Results:**

Mean SSB intake was 36·3 (sd 42·5) fluid ounces or 433·4 (sd 493·6) calories per day. In the final step of the regression model, seven variables significantly explained adolescent’s SSB consumption: behavioural intention (*P* < 0·05), affective attitude (*P* < 0·05), perceived behavioural control (*P* < 0·05), health literacy (*P* < 0·001), caregiver behaviours (*P* < 0·05), caregiver rules (*P* < 0·05) and home availability (*P* < 0·001).

**Conclusions:**

SSB intake among adolescents in rural Appalachia was nearly three times above national mean. Home environment was the strongest predictor of adolescent SSB intake, followed by caregiver rules, caregiver behaviours and health literacy. Future interventions targeting these factors may provide the greatest opportunity to improve adolescent SSB intake.

Adolescence, aged 12–19 years, is a transitional period and health habits developed during this time often continue into adulthood^(1,2)^. Addressing and encouraging healthy dietary habits, including limiting sugar-sweetened beverages (SSB), are especially important during adolescence^(2)^. SSB include sweetened fruit flavoured drinks, regular soda or soft drinks, energy drinks, sports drinks, and sweetened coffees and teas^(3)^. Excessive consumption of SSB has been linked to multiple health concerns such as obesity, obesity-related cancers, type 2 diabetes, CVD and dental caries^(4,5)^.

Within the USA, SSB are substantial sources of increased calories and added sugar within the diets of adolescents^(4,6)^. Among US adolescents, about 63 % consume at least one SSB per day and SSB contribute a mean of 143 calories per day^(6)^. Adolescents are the highest consumers of SSB with more than 9 % of their total daily calories attributed to SSB consumption^(6)^, with SSB consumption highest among adolescent males and increasing with age^(6,7)^. Regional disparities in adolescent SSB intake are also evident, especially in rural Appalachia, the targeted region of this research^(8,9)^. Adolescents within rural Appalachia have disproportionately high intakes of SSB^(10–12)^. More specifically, one regional study indicated mean SSB intake among middle school adolescents was about 457 calories per day, nearly three times the national mean^(10–12)^. Along with SSB concerns, this region suffers from high prevalence of SSB-related chronic health conditions (e.g., obesity, poor oral health) and faces substantial barriers to health, economic and social equality, such as lack of access to medical and preventative services, financial struggles, lack of health insurance, transportation issues, geographical isolation and food insecurity^(8,9)^. Other Appalachian focused studies indicate important socio-cultural influences may account for higher SSB intake among adolescents, such as peer influence of cultural norms, being resistant to change or accepting help, matriarchal food gatekeeper and strong family ties^(8,9)^.

With growing health concerns related to high US consumption of SSB, interventions and strategies have been developed with foundations in the socio-ecological model (SEM). The SEM is centred on highlighting interdependence, or lack thereof, of internal and external factors that influence an individual’s behaviours^(13,14)^. According to SEM, an individual’s behaviour is influenced by intrapersonal (e.g., knowledge, attitudes, self-concept), interpersonal (e.g., social norms, family, peers) and environmental factors (e.g., home environment, community, public policy) ^(14)^. Intervention strategies that have applied multi-level SEM approaches have shown improvements related to SSB intake, healthy eating patterns, physical activity and childhood obesity^(13,15,16)^.

Specific to SSB, studies that have incorporated single-level approaches, such as focus on intrapersonal level factors, have shown constructs such as subjective norms, perceived behavioural control and media literacy play crucial roles in an adolescent’s intentions to consume SSB^(17–22)^. Constructs from the theory of planned behaviour (TPB) and health literacy concepts are intrapersonal variables associated with SSB intake. TPB constructs include behavioural intention, affective and instrumental attitudes, subjective norms and perceived behavioural control^(23)^. Health literacy concepts are often referred to as a general skill set (i.e., the ability to obtain, find, understand and use information to make health decisions) ^(24–26)^. On the contrary, health literacy concepts are sometimes considered in specific domains (i.e., media literacy as the ability to access, analyse, process and produce media messages; public health literacy as the ability to obtain, interpret and act on information needed to make decisions that benefit the health of a community) ^(26,27)^. Collectively, these concepts refer to an adolescent’s ability to internally process and understand factors contributing to their own health outcomes and the health of their community^(24–27)^.

In other single-level studies on either interpersonal or environmental factors, evidence shows caregiver behaviours, caregiver practice and home availability are important factors influencing adolescent’s SSB intake^(28–30)^. In addition, a recent observational and cross-sectional study applied an SEM approach to explore factors contributing to SSB intake among a nationally representative sample of US adolescents^(16)^. Relative to intrapersonal and other social factors examined, caregiver practices and home availability showed the strongest influence on adolescent SSB behaviours, since these factors provide social support and behaviour modelling, as caregivers are usually the gatekeeper of food and beverages within the home and set an example for their adolescent about what are healthy or unhealthy choices and help develop the adolescent’s perceptions related to SSB^(13,16,19,28,30–35)^. Among adolescents, home SSB environment and availability is one of the most influential predictors of food and beverage choices, and SSB behaviours^(36,37)^. Adolescents with home access to SSB are twice as likely to be moderate consumers of SSB and five times more likely to be high consumers of SSB^(38)^. Furthermore, about 55–70 % of all SSB consumed by adolescents were consumed in the adolescent’s home^(39,40)^.

However, current literature is limited regarding how multiple levels of the SEM concurrently influence an adolescent’s consumption of SSB. Understanding these impacts within health disparate regions with excessive SSB intake, like rural Appalachia, is necessary to inform design and implementation of health promotion programmes. This cross-sectional study addresses gaps in literature by targeting a regional sample of Appalachian adolescents with the primary aim of identifying SEM factors associated with adolescents’ SSB intake, while controlling for relevant demographic factors. SEM levels targeted in the current study included intrapersonal factors (i.e., attitudes, subjective norms, perceived behavioural control, behavioural intentions, health literacy, media literacy, public health literacy), interpersonal factors (i.e., caregiver’s SSB behaviours, caregiver’s SSB rules) and environment (i.e., home SSB availability).

## Methodology

### Study design

This cross-sectional study is a secondary analysis of data from the Kids SIP*smart*ER trial targeting Appalachian middle school students and their caregivers. The Kids SIP*smart*ER intervention is a school-based, behaviour and health literacy programme aimed at improving SSB behaviours among seventh grade middle school students and engages caregivers in SSB role modelling and supporting home SSB environment changes. Kids SIP*smart*ER is grounded by TPB constructs and health literacy, media literacy, numeracy and public health literacy concepts. Evaluation of effectiveness and implementation of the multi-level Kids SIP*smart*ER intervention across twelve Appalachian middle schools through a type 1 hybrid design and cluster randomised controlled trial is on-going (Clincialtrials.gov: NCT03740113; 2018–2022) ^(41,42)^. The current study utilises baseline data from eight middle schools enrolled in the first 2 years of the study.

### Study setting

For this cross-sectional analysis, four Appalachian counties are represented. As indicated by scores on the United States Department of Agriculture Rural-Urban Continuum Code (1 = urban, 9 = rural), the included counties are mostly rural: Buchannan = 9, Smyth = 7, Tazewell = 5 and Wythe 6^(43)^. According to school-level data, over 90 % of all adolescents meet on-time graduation rates, despite chronic absenteeism reaching between 11 and 22 %^(44)^. Finally, state assessments in these counties for reading range from 72 to 86 %, math range from 79 to 91 % and science range from 77 to 90 %^(44)^.

### Eligibility and recruitment

To be eligible for inclusion, schools had to be in the geographical Appalachian region, have approximately 80–200 adolescents enrolled in seventh grade and have eighth grade within the same school building as seventh grade^(41)^. Within each school, all seventh grade adolescents were eligible to participate. Specific to this secondary analysis, only students with complete data were included.

At each school, an informational letter signed by the school’s principal, a study flyer and consent form were sent home to the adolescent’s caregivers^(41)^. Additional strategies at some schools included members of the research team attending ‘Back-to-School Nights’, addressing individual classes within each school about the programme, or personalised phone calls to caregivers to obtain verbal consent or remind caregivers to return the consent forms^(41)^. Recruitment efforts for caregivers and adolescents were customised to the needs of each school and based on a combination of strategies to increase response rates. Adolescent participation in the current study required provision of both parental consent and adolescent assent. Teachers assisted with distributing, collecting and following up with adolescents for caregiver consent forms. Adolescents who returned the signed consent form (permission granted or denied) received a nominal prize (e.g., highlighter). The assent procedure was conducted immediately before data collection. An assent statement was read with adolescents and all questions were answered before signature was obtained^(41)^.

Adolescents were administered the validated survey instrument at baseline during one class period (approximately 45 min). One researcher read each question and answer option aloud to the entire class, while 1–3 additional research staff answered individual student questions (staffing dependent on size and structure of classroom).

### Measures

Demographic characteristics included variables of gender and age. Scaled response options are illustrated in Tables 1 and 2. All anchors for the Likert scales used can be found in Table 3.

**Table 1:** Bivariate associations between intrapersonal level variables and adolescent sugar-sweetened beverage (SSB) intake (*n* 793)

Variables	*n*	%	Total ounces SSB consumed/d		*F*-statistic	*P*
			Mean^c^	sd		
Student behavioural intentions						
Negative	439	55·4	43·2^a^	47·9	13·7	< 0·001
Neither positive nor negative	122	15·4	30·9^b^	33·9		
Positive	232	29·3	26·2^b^	32·3		
Student affective attitudes						
Unenjoyable	329	41·5	31·0^a^	35·3	21·3	< 0·001
Neither enjoyable nor unenjoyable	191	24·1	26·8^a^	32·1		
Enjoyable	273	34·4	49·4^b^	52·6		
Student instrumental attitudes						
Unhealthy	559	70·5	35·3^a^	39·5	3·8	< 0·05
Neither healthy nor unhealthy	116	14·6	31·9^a^	37·9		
Healthy	118	14·9	45·8^b^	57·0		
Student subjective norms						
Disagree	260	32·8	45·3^a^	47·0	8·9	< 0·001
Neither agree nor disagree	219	27·6	33·3^b^	38·3		
Agree	314	39·6	31·1^b^	40·3		
Student perceived behavioural control						
Disagree	153	19·3	52·5^a^	51·9	14·6	< 0·001
Neither agree nor disagree	87	11·0	35·8^b^	42·6		
Agree	553	69·7	31·9^b^	38·4		
Health literacy						
High likelihood of limited literacy	359	45·3	41·7^a^	48·8	5·8	< 0·01
Possibility of limited literacy	290	36·6	33·5^b^	35·7		
Adequate Literacy	144	18·2	28·8^b^	36·3		
Media literacy						
Disagree	110	13·9	41·2	45·0	0·9	> 0·05
Neither agree nor disagree	289	11·2	34·8	37·7		
Agree	394	49·7	36·1	45·0		
Public health literacy						
Disagree	511	64·4	37·7	44·7	1·4	> 0·05
Neither agree nor disagree	246	31·0	34·7	39·1		
Agree	35	4·4	26·5	30·0		

^a,b^One-way ANOVA tests were used to assess if there were any significant differences between the means of the categories. Post-hoc analyses were done using the Tukey method. Values without the same superscript letter are significantly different (*P* < 0·05).

^c^For each of the five SSB questions (i.e., regular soft drinks, sweetened juice beverage/drink, sweetened tea, coffee with sugar, energy drinks), adolescents report consumption frequency across seven response categories (ranging from never or < 1 time per week to three or more times per day) and reported portion sizes across six response categories (ranging from 6 ounces or less to greater than 20 ounces). Total SSB was derived using standardised and validated scoring procedures.

**Table 2:** Bivariate associations between interpersonal and environmental level variables and adolescent sugar-sweetened beverage (SSB) intake (*n* 793)

Variables	*n*	%	Total ounces SSB consumed/d		*F*-statistic	*P*
			Mean^c^	sd		
Caregiver rules						
Disagree	421	53·1	43·7^a^	44·7	14·5	< 0·001
Neither agree nor disagree	305	38·5	29·1^b^	40·1		
Agree	67	8·4	23·2^b^	28·1		
Caregiver behaviours (caregiver SSB intake)						
Frequent consumer	117	14·8	30·2^a^	41·3	6·0	< 0·01
Sometimes consumer	261	32·9	31·2^a^	41·1		
Rare consumer	415	52·3	41·3^b^	43·2		
Home availability of SSB						
Rarely available	289	36·4	23·3	31·1	57·2	< 0·001
Sometimes available	379	47·8	35·6	34·5		
Frequently available	125	15·8	68·8	64·8		

^a,b^One-way ANOVA tests were used to assess if there were any significant differences between the means of the categories. Post-hoc analyses were done using the Tukey method. Values without the same superscript letter are significantly different (*P* < 0·05).

^c^For each of the five SSB questions (i.e., regular soft drinks, sweetened juice beverage/drink, sweetened tea, coffee with sugar, energy drinks), adolescents report consumption frequency across seven response categories (ranging from never or < 1 time per week to three or more times per day) and reported portion sizes across six response categories (ranging from 6 ounces or less to greater than 20 ounces). Total SSB was derived using standardised and validated scoring procedures.

**Table 3:** Stepwise regression model to explain adolescent sugar-sweetened beverage (SSB) intake using factors across the socio-ecological model (*n* 793)

		Step 1: Demographic factors		Step 2: Intrapersonal level factors		Step 3: Interpersonal level factors		Step 4: Environmental level factors	
Variables	Scale descriptions	Avg ME	Robust SE	Avg ME	Robust SE	Avg ME	Robust SE	Avg ME	Robust SE
Demographics									
Gender	1 = Male, 0 = Female	+7·2	3·0*	+4·4	3·3	+6·1	3·4	+5·1	3·4
Age	1 = 11 years old, 2 = 12 years old, 3 = 13+ years old	+9·4	2·9**	+6·6	2·8*	+6·5	2·8*	+6·4	3·4
Intrapersonal									
Behavioural intention	Range between 1 and 7 with larger value means Extremely Positive	**–**		−3·3	0·9***	−2·5	0·7***	−1·5	0·7*
Affective attitude	Range between 1 and 7 with larger value means Extremely Enjoyable	**–**		−2·8	1·0**	−2·5	1·0**	−2·1	0·9*
Instrumental attitude	Range between 1 and 7 with larger value means Extremely Healthy	**–**		−0·1	0·7	−0·1	0·7	−0·0	0·8
Subjective norms	Range between 1 and 7 with larger value means Strongly Agree	**–**		−0·9	0·8	−0·5	0·8	−0·7	0·7
Perceived behavioural control	Range between 1 and 7 with larger value means Strongly Agree	**–**		−1·0	0·5	−0·8	0·5	−1·20·5*	
Health literacy	Range between 1 and 6 with larger value means Adequate health literacy	**–**		−4·1	1·3**	−4·1	1·3**	−4·0	1·1***
Media literacy	Range between 1 and 33 with larger value means Strongly Agree	**–**		+1·3	0·8	+1·5	0·8	+0·1	1·1
Public health literacy	Range between 1 and 18 with larger value means Strongly Agree	**–**		−0·20	2·3	+0·2	2·5	+1·1	2·3
Interpersonal									
Caregiver behaviours	Range between 1 and 5 with larger value means Never	**–**		**–**		−5·1	1·3***	−3·4	1·4*
Caregiver rules	Range between 1 and 36 with larger value means Strongly Agree	**–**		**–**		−5·6	2·4*	−4·6	2·3*
Environmental									
Home availability	Range between 1 and 21 with larger value means Always	**–**		**–**		**–**		+15·1	1·4***


Avg ME: average marginal effects; robust SE: cluster robust standard errors.

*Significant at *P* < 0·05, **P < 0·01, ***P < 0·001.

#### Sugar-sweetened behaviours

An adapted version of the validated Beverage Intake Questionnaire was used to assess the dependent variable, adolescent SSB behaviours^(3,45,46)^. The Beverage Intake Questionnaire-15 focuses on frequency and portion sizes of fifteen beverage categories. To meet the needs of the current study, alcohol items were removed and the three types of milk were consolidated into a single milk category, resulting in a ten-item assessment. Importantly, the five items necessary to compute amounts of SSB (i.e., regular soft drinks, sweetened juice beverage/drink, sweetened tea, coffee with sugar, energy drinks) were not altered. For each SSB question, adolescents report consumption frequency with seven response categories ranging from never or < 1 time per week to 3 or more times per day. Portion sizes were also reported across six response categories ranging from 6 ounces or less to > 20 ounces. Using standardised and validated scoring procedures, daily totals for each type of SSB were totalled by multiplying intake frequency of the SSB category by the portion reported for that SSB category^(3,43,45)^. Likewise, the five categories of SSB were summed to obtain total intake of all SSB per day.

#### Intrapersonal variables

Intrapersonal level variables included TPB constructs, health literacy, media literacy and public health literacy. TPB constructs were assessed on a seven-point Likert scale and included behavioural intention (two items), affective attitude (one item), instrumental attitude (one item), subjective norms (one item) and perceived behavioural control (one item) ^(10,12,19)^.

Health literacy was assessed with the six-item Newest Vital Sign. Using validated procedures, scores were categorised on total score values: 0–1 indicates high likelihood of limited health literacy, 2–3 indicates the possibility of limited health literacy and 4–6 designates adequate health literacy^(47–49)^. Media literacy was assessed as the mean of six items measured on a seven-point Likert scale (Cronbach’s *α* = 0·59)^(10,26)^. Public health literacy was measured as the mean of four items assessed on a five-point Likert scale (Cronbach’s *α* = 0·68)^(10,26,50)^.

#### Interpersonal variables

Interpersonal level variables were measured on a five-point Likert scale and included one item on caregiver’s SSB behaviours and ten items on caregiver’s rules around adolescent SSB consumption (Cronbach’s *α* = 0·66) ^(34,51)^.

#### Environmental variable

Reported on a five-point Likert scale, environmental level variables assessed home availability of SSB with the same five SSB item reported for total SSB adolescent consumption (i.e., regular soft drinks, sweetened juice beverage/drink, sweetened tea, coffee with sugar, energy drinks) (Cronbach’s *α* = 0·51)^(34,51)^.

### Analyses

SPSS version 26.0 was used for summary statistics and ANOVA analyses, while Stata 16.0 was used for regression analysis. For independent variable measures including more than two questions, Cronbach’s *α* values were conducted to determine internal consistency of scales. Although several constructs in our model were measured with multiple questions, there are no consistent methods in the literature in terms of how to aggregate variables into one single index or multiple ones. To serve our purpose of identifying the impacts of categories of SEM, we used the mean scores across multiple questions as the level of those constructs with more than one variable information collected. The procedure of averaging those Likert scale variables resulted in continuous-like values for those constructs and was treated as continuous variables in the interpretation of regression model results.

In the ANOVA analysis, we rounded the mean construct level values into the nearest appropriate Likert category to create Likert scale at the construct level (e.g., on a 5-point Likert scale 3–3·99 = neither agree nor disagree, 4–4·99 = somewhat agree, etc.). ANOVA were completed to identify bivariate associations between SSB consumption within our adolescent sample and demographic characteristics, intrapersonal, interpersonal, environmental and exploratory variables. Tukey’s test of significance level at *P* ≤ 0·05 determined statistically significant post hoc relationships. We conducted ANOVA using the full five- or seven-point Likert scale range for each variable, and by consistently collapsing into three category response options. Overall statistical and post hoc interpretations were remarkably similar, so the reduced option is presented.

Gender, age, intrapersonal, interpersonal and environmental variables were entered into a modified two-part model in a stepwise fashion^(52,53)^. Our sample contains modest numbers of zero SSB consumption reported by adolescents. These ‘zeros’ are true zero instead of missing or censoring, and they cause sizeable skewness to the SSB outcome distribution. Therefore, we choose the modified two-part model that generalises the Tobit model to analyse those data with true zeros and is more robust to distribution assumptions^(53,54)^. The two-part models we estimated contain the first part that handles the nonlinear process of generating consume *v*. not-consume decisions as probit model and the second part that estimates the nonzero continuous SSB consumption via a log-link generalised model and the se are adjusted to be school-year cluster robust. To ensure comparability across models, we restricted the sample size to be the same across all models (i.e., the smallest set of observations that have non-missing values across all the variables in the largest model specification in step 4). Following the conceptual framework of Kids SIP*smart*ER^(41)^, independent variables were included based on their hypothesised proximal influence on SSB consumption (i.e., behavioural intention most proximal, home environment most distal): step 1: demographic factors, step 2: intrapersonal variables, step 3: interpersonal variables and step 4: environmental variables.

## Results

### Participants

Within the eight schools, 1360 seventh grade adolescents were eligible to participate. After caregiver consent and adolescent assent processes, 874 adolescents were consented, 862 (63 %) adolescents completed the baseline survey and 793 (58 %) were included in the current study. The seventy-two adolescents not included in the analysis had missing data for the variables evaluated. Gender distribution was relatively equal with 55·4 % females and 44·6 % males. Age distribution of adolescents included 3·9 % aged 11 years, 80·5 % aged 12 years and 15·7 % aged 13 or older.

### Bivariate association between adolescent sugar-sweetened behaviour intake and socio-ecological model factors

Across all students, SSB intake was a mean of 36·3 (sd = 42·5) ounces and 433·4 (sd = 493·6) calories per day. Compared with reported SSB ounces per day among females (32·2, sd = 34·6), males consumed significantly more SSB ounces per day (41·5, sd = 50·3) (*P* = 0·002). Reported SSB ounces per day increased with each year older from 11 years of age (32·9, sd = 29·6), 12 years of age (34·3, sd = 40·1) to 13 years of age or older (47·6, sd = 54·2) (*P* = 0·006).

As illustrated in Tables 1 and 2, all intrapersonal, interpersonal and environmental factors were associated with SSB intake in the direction hypothesised (all *P* < 0·05). For example, higher affective attitudes, instrumental attitudes, subjective norms, perceived behavioural control, behavioural intentions, health literacy, media literacy and public health literacy were associated with less reported SSB ounces per day (all *P* < 0·05). Similarly, as adolescents reported stronger agreement with caregiver SSB rules and more positive caregiver SSB behaviour, the reported SSB ounces per day was lower (both *P* < 0·01). Finally, as adolescents reported more prominent availability of SSB within their home environment, the reported SSB ounces per day was higher (*P* < 0·001).

### Stepwise regression results

Table 3 presents the associated average marginal effects and cluster robust se of those marginal effects for variables in each step of the stepwise modelling process. In the final step, seven variables show statistically significant contribution in explaining adolescent’s SSB consumption: behavioural intention, affective attitude, perceived behavioural control, health literacy, caregiver behaviours, caregiver rules and home availability. For each 1-unit increase in behavioural intentions and affective attitudes, adolescents had a mean reduction in SSB consumption at the rate of about 1·5 ounces and 2·1 ounces per day, respectively (*P* < 0·05). Perceived behavioural control was also statistically significant at 5 % significance level and is similarly interpreted: adolescents had a mean SSB intake reduction by about 1·2 ounces per day for each 1-unit increase in perceived behavioural control. For every 1-unit increase in health literacy, adolescents showed a mean of 4·0-ounce decrease in SSB consumption per day (*P* < 0·001). Related to interpersonal and environmental factors, all three variables showed statistically significant influences in adolescents’ SSB daily consumption. For each 1-unit increase in caregiver behaviours and caregiver rules, there was a mean of 3·4-ounce and 4·6-ounce decrease in SSB intake per day (*P* < 0·05), respectively. Related to the environmental level variable, for each 1-unit increase in home availability there is a mean of 15·1-ounce increase in adolescents’ SSB consumption per day (*P* < 0·001). This large magnitude of the net influence, as compared with other constructs, signals the clinical significance of home availability.

Steps 1 through 3 of the regression models also reveal notable findings. For demographic factors, gender and age were both significant in step 1. More specifically, male adolescents on average consumed 6·9 more ounces of SSB per day relative to female adolescents and age accounted for an additional 9·3 ounces of SSB for each 1-unit increase of age in years. However, gender was no longer significant after including intrapersonal factors. Age remained significant when including both intrapersonal and interpersonal factors, but was no longer significant when environmental factors were included. For TPB constructs, behavioural intentions and affective attitudes were significant in each step, yet the marginal effects decreased with the addition of interpersonal and environmental factors which is to be expected since inclusion of those additional factors resulting in the net effect of TPB constructs (i.e., netting the influence of interpersonal and environmental factors that overlapped with TPB). Instrumental attitudes and subjective norms were not significant in any steps, while perceived behavioural control was only significant in the final step 4 of the model. Health literacy was significant across each step, yet neither media literacy nor public health literacy significantly contributed to the model. Caregiver behaviours and caregiver rules related to SSB were significant in step 3 and remained significant, although the marginal effects somewhat decreased, with the addition of the environmental variable in step 4.

## Discussion

Our study fills an important gap in demonstrating how multiple levels of SEM influence rural Appalachia adolescents who are disproportionately burdened with numerous health disparities impacted by excessive SSB intake^(55,56)^. Our study showed rural Appalachia adolescents consumed a mean of 439 calories per day, which is similar to other research in this region^(6,10,11)^. This is excessively higher than recommended daily added sugar intake of < 10 % of daily caloric intake and over 300 % higher than national mean adolescent SSB intake^(6,10,11)^. When including all SEM levels within our stepwise regression to explain adolescent’s SSB intake, the marginal effects and level of significance were strongest from home availability, followed by caregiver rules, health literacy, caregiver behaviours, affective attitudes, behavioural intentions and behavioural control.

Within other literature, home availability of SSB has shown to be one of the strongest factors influencing adolescent SSB intake^(31,36,38–40,57,58)^. Similar to our findings, studies have shown significant associations with increased adolescent SSB intake when SSB are more readily available in the home; however, few have evaluated home availability of SSB in conjunction with other variables (e.g., interpersonal, intrapersonal and macro-level factors) ^(28,35,38,40)^. Another qualitative study among adolescents highlights the important influence of home SSB availability on adolescent recognition of SSB intake at home and norms around availability in the home^(58)^. However, a large portion of the intervention literature does not address the influence of home SSB availability. For example, one systematic review evaluated fifty-five interventions targeting child and adolescent SSB intake and found only four studies addressed the adolescent’s home environment^(35)^. Collectively, these findings suggest the importance, yet limited intervention approaches, of targeting home environment with efforts focused on improving adolescent SSB behaviours.

Similar to home environment, interpersonal level factors that included caregiver rules and caregiver behaviours also explained adolescent SSB intake. Our finding supports prior literature that demonstrates the important association among caregiver behaviours and adolescent SSB intake^(29,31–34,36)^. Although fewer studies have evaluated caregiver rules, some showed caregivers who take a role in active guidance could help reduce adolescent SSB intake^(31,36)^. Other studies further illustrate how additional caregiver traits, such as education level and attitudes towards SSB, can influence adolescent SSB behaviours^(31,33,36)^. While published findings related to the important role of caregiver rules and behaviours on adolescent SSB intake are consistent, most research in this area is cross-sectional. There is limited evidence of interventions that have successfully targeted caregivers’ rules and behaviours to help improve their adolescents SSB intake. Given limited evidence of interventions having successfully targeted parents’ rules and behaviours to help improve their adolescent’s SSB intake, this is an important focus for future research.

While our findings suggest environmental and interpersonal factors strongly predict adolescent SSB intake within rural Appalachia, four intrapersonal factors also significantly contributed to the final model. Three of these intrapersonal factors were related to TPB: behavioural intention, affective attitudes and perceived behavioural control. The important influence of behavioural intention and perceived behavioural control on adolescent SSB behaviour in our study is consistent with previous TPB literature^(10,12,19,20,59,60)^. Interestingly, the TPB construct with the greatest influence on adolescent SSB intake in our study was affective attitude (i.e., emotional reaction to the outcome of a behaviour) ^(23)^. Although subjective norms were significant in the ANOVA analysis, it was not significant in the regression model when accounting for all other constructs. This suggests that the perceived influence of the friends on adolescents SSB behaviours is not as important as other included factors. The fourth significant intrapersonal factor in the final model was health literacy. This finding is reflective of existing literature that suggests health literacy is one of the strongest intrapersonal predictors of health status and outcomes in adults^(24)^. While less frequently studied in adolescents, our study affirms findings from a previous study suggestion that lower health literacy scores were associated with higher SSB intake^(61)^. While neither media literacy nor public health literacy contributed to the final model, we believe they warrant further investigation in future studies to understand their impact on SSB intake. These factors were individually associated with SSB intake in the ANOVA tests and reflect factors known to influence SSB intake and other health behaviours (i.e., marketing exposure, perceptions of supporting the health of their community) ^(16,50,62–65)^.

In step 1 of our regression model, when only considering demographics, males consumed significantly higher amounts of SSB relative to females and older adolescents consumed significantly more SSB than their younger counterparts. These gender and age findings are similar to existing literature^(6)^. Yet these demographic factors no longer remained significant when adding higher-level SEM variables of influences. Collectively, our findings underscore the importance of each SEM level to understand adolescent SSB intake and in the design and evaluation of interventions targeting adolescent SSB intake.

### Limitations and strengths

Several study limitations should be considered. First, all variables within our study were self-reported by the adolescents and could be subject to self-report bias. Similarly, responses related to caregiver SSB rules and SSB behaviours were the adolescent’s perceptions of their caregivers. There may be discrepancies with the perceived caregiver actions as reported by adolescents *v*. actual caregiver actions. Second, student demographic data related to race and ethnicity were collected but not included in our analysis due to inconsistencies between self-reported and census data for southwest Virginia region of Appalachia. It was concluded that questions related to race and ethnicity were unfamiliar knowledge to most adolescents, leading to inaccurate report. Yet, given limited diversity in this region (95·1 % White and 98·8 % non-Hispanic), the implications of not including race and ethnicity variables are postulated to have little impact on model interpretations or study conclusions^(44)^. Third, since our study was cross-sectional in design, cause and effect cannot be determined. Fourth, Cronbach’s *α* values for media literacy and public health literacy were on the low end of satisfactory for internal consistency and should be interpreted somewhat cautiously.^(66)^ Finally, due to targeted rural status and unique cultural norms within southwest Virginia related to SSB intake, the current study may lack generalisability to other regions^(10,11)^. These limitations should be considered within the study strengths, including strong theoretical approach, use of previously validated questionnaires, standardisation in survey administration and an adequate sample across the eight schools and four counties in the rural, health disparate Appalachia region.

### Implications for future research

Based on findings from our cross-sectional study, future research should include more robust intervention approaches focused on a multi-level SEM approach to improve SSB intake for adolescents with an emphasis on home availability, caregiver influences and personal influences (i.e., behavioural intentions, affective attitudes, perceived behavioural control and health literacy). Importantly, the on-going Kids SIP*smart*ER intervention trial is filling this gap by focusing on these levels of influence with a school-based curriculum targeting SSB behaviours among seventh grade middle school students and an integrated text messaging programme targeting caregivers^(41)^. The 6-month, 12-session intervention curriculum is guided by TPB constructs and health literacy concepts and applies numerous evidence-based behavioural change techniques to target adolescents SSB behaviour change. For caregivers who consent to participate in the text message component, the content aligns with the adolescents’ curriculum, with assessments every 5–6 weeks, in which caregivers select personal, interpersonal or environmental barriers and receive targeted strategy messages focused on these factors. These strategies offer tips and techniques guiding caregivers to decrease their own SSB intake, as well as improve SSB-related caregiver practices, rules and home environment. Future findings from the multi-level Kids SIP*smart*ER intervention trial will help to identify causal factors influencing high SSB intake among rural Appalachian adolescents, as well as verify the findings from this cross-sectional analysis.

## Conclusion

Our study identifies home environment as the strongest predictor for adolescent SSB intake within rural Appalachia across all levels of the SEM. There is also strong evidence of the influence caregiver rules and behaviours have on adolescent SSB intake, and various other intrapersonal factors including adolescent health literacy and behavioural intentions. Thus, our study adds to the literature by identifying home environment and caregiver rules and behaviours as the most influential factors to adolescent SSB intake. The use of these findings can help develop further research in the area of multi-level health interventions that help reduce SSB behaviours for adolescents, especially those targeting home environment and caregivers. Given the negative influence of SSB on adolescent health, especially in rural Appalachia, but also nationally, there is need for further understanding about various levels of influence so effective interventions can be developed and implemented.
